# Moderators of Social Facilitation Effect in Virtual Reality: Co-presence and Realism of Virtual Agents

**DOI:** 10.3389/fpsyg.2020.01252

**Published:** 2020-06-15

**Authors:** Paweł M. Strojny, Natalia Dużmańska-Misiarczyk, Natalia Lipp, Agnieszka Strojny

**Affiliations:** ^1^Faculty of Management and Social Communication, Institute of Applied Psychology, Jagiellonian University, Kraków, Poland; ^2^R&D Unit, Nano Games sp. z o.o., Kraków, Poland

**Keywords:** social facilitation, co-presence, social presence, virtual reality, audience effect

## Abstract

Social facilitation has been researched for decades, but in the face of the development of virtual reality technology, new questions arise regarding the possibility of its occurrence in this environment —in the presence of computer-generated agents. Past research provided inconclusive answers: several experiments confirmed this possibility, but several others disagreed. On the other hand, previous studies have shown the important role of VR characteristics, such as realism or co-presence, in evoking other psychological phenomena. However, no study has investigated the interplay between the presence of computer-generated agents and perceived social realism in evoking social facilitation in virtual reality. To this end, the present randomized control study was conducted. The sample consisted of professional firefighters (*N* = 48), divided into an experimental group with virtual bystanders and a control group without them. Subjects were instructed to perform a rescue procedure in a virtual reality headset. The performance of participants was logged and they completed questionnaires regarding sense of presence in the virtual environment, perceived realism of the environment and perceived co-presence of virtual agents. The obtained results confirmed the role of social realism as a moderator of the occurrence of social facilitation in the presence of computer-generated agents. At the same time, the main effect of facilitation was not confirmed. These results support predictions that the subjective feeling of being in a realistic company of others may be more important in evoking social facilitation than objective facts. Furthermore, the results contribute to the debate regarding the mechanism of social facilitation, suggesting that simple augmentation of the environment with social distractors is not always enough, thus questioning the attentional explanation of the effect. Taken together, our results extend previous findings on social facilitation and open up new possibilities for designing effective virtual environments.

## 1. Introduction

The influence of other people on individuals performing a task is a common problem in real life. For example, bystanders are often present at various accident sites, possibly influencing performance of the rescuers. It is important to try to understand this influence and studying it in terms of the social facilitation effect appears to be a promising direction. Moreover, if such influence can be replicated in a virtual environment, rescuers (or other people exposed to the influence of bystanders during their work) could be trained in conditions similar to those which are present in real life. In the present paper, results of a study on social facilitation in virtual reality, specifically—in a rescue context, will be described.

Social facilitation occurs “when one animal increases or decreases its behavior in the presence of another animal which does not otherwise interact with it” (Guerin, 2010). Performance is improved for easy tasks (facilitation) and deteriorated for difficult ones (inhibition). For more detailed description of social facilitation see former works (Zajonc, 1965; Bond and Titus, 1983; Baron, 1986). Although this phenomenon has been known for a long time in psychology (Triplett, 1898), researchers are still far from full understanding of it (Cottrell, 1972; Baron, 1986; Huguet et al., 1999). One of several theoretical controversies particularly relevant to current study regards the issue of the *mere presence* vs. *audience* to trigger the social facilitation effect. According to early definition, the sufficient condition of social facilitation occurrence is the presence of others, even if the actor is not an object of their interest (Zajonc, 1965). On the other hand, further studies showed that it is not enough—others have to be focused on the actor (Cottrell et al., 1968). Because the current study was set in a VR depicting specific task (rescue action), the presence of victims (and no bystanders) was necessary in both conditions due to the ecological validity. Being aware of the controversy mentioned earlier, we decided to manipulate with the presence of others being able to observe an actor (bystanders) assuming the presence of victims will not cause the studied effect since they are preoccupied thus unable to observe the actor.

With the development of virtual reality (VR) technologies, researchers have begun to explore the impact of computer-generated agents in virtual environments (VEs) on users in terms of the social facilitation effect. Several studies examining this phenomenon in VR have been published, but according to the recent review their results are not consistent (Sterna et al., 2019). To the best of our knowledge, the full social facilitation and inhibition effect in easy and difficult tasks respectively has been shown only once in VR (Park and Catrambone, 2007). The possibility of its occurrence is supported by the results of other studies in which only social facilitation took place (Pan and Hamilton, 2015; Murray et al., 2016). In several other studies social inhibition was observed (Hoyt et al., 2003; Zanbaka et al., 2007; Emmerich and Masuch, 2016). However, other studies report a null effect (Hayes et al., 2010; Baldwin et al., 2015; Pan and Hamilton, 2015). This discrepancy may stem from methodological shortcomings, but it is possible that other unrevealed variables moderate the relationship. The moderator may affect the direction and/or strength of the relation between dependent and independent variables. We believe that Co-presence, Sense of Presence and some aspects of Realism may play a role here since they are related to subjective impression of being among others in virtual reality. Because the feeling of being in the company of others plays a pivotal role in social facilitation effect, we believe that these variables are able to affect the strength (but not the direction) of this relationship (not necessarily lowering it to zero given the subjective nature of moderators being proposed).

Previous studies have proved the importance of perceived presence (*sense of presence*, defined as the “sense of being there” in a virtual environment, or a human reaction to the experiences the technology delivers; Slater, 2003) in evoking desired reactions to VR (Poeschl and Doering, 2014; Riva et al., 2014). However, in the light of a recent meta-analysis it is also possible that integrating different factors of the multidimensional construct of sense of presence into a single score may be unable to capture the key characteristics responsible for evoking these reactions (Ling et al., 2014). Moreover, realism, defined as the fidelity of simulation—how accurate is the replication of the real environment and objects in virtual reality (Bowman and McMahan, 2007; Poeschl and Doering, 2013) may also play a role in one's responses to a virtual environment. Perhaps the social aspects of realism (understood as impression of fidelity of agents located in VR) play a key role here, particularly in case of phenomena closely related to social interactions, such as social facilitation. Another variable which might be of interest when trying to understand human reactions in a virtual environment, is co-presence—the impression of being in the environment with others, even when they are not physically present and even when they are not humans, but computer-generated agents (Youngblut, 2003). It has been proved that it may be crucial for evoking desired reactions to socially interactive VR (Poeschl, 2017; Felnhofer et al., 2019). Based on the past results, one might expect realism (in the social aspects in particular), sense of presence and co-presence to affect the occurrence of the social facilitation effect, but researchers have not yet controlled these variables.

Summarizing, to shed more light on the relationship between subjectively assessed social characteristics of VR (co-presence, sense of presence, realism) and social facilitation, we conducted a study in which for the first time to our knowledge the level of these characteristics was controlled. It was done in order to determine if they moderate the occurrence of the social facilitation effect evoked by computer-generated agents in VR. The presented study was preceded by an exploratory one, in which we found an interactive influence of spectators' presence and realism on subjectively assessed performance. To capture the social facilitation effect in terms of objective performance, we conducted the study described herein. Since the task we used was well-known by the participants (emergency procedure, professional fire-fighters), we expected the social facilitation (not inhibition) effect. We hypothesized that co-presence (Hypothesis 1), sense of presence (Hypothesis 2), and realism (Hypothesis 3) would moderate the relationship between audience presence and performance: high level of co-presence, sense of presence, and realism separately would be a condition of occurrence of social facilitation, while low level would not.

## 2. Materials and Methods

### 2.1. Participants

Participants were recruited at the College of the State Fire Service and firefighting units in Cracow (Poland); all of them had undergone at least one year of training and had participated in real-life rescue operations. This research was accepted by the Ethical Committee at Jagiellonian University Institute of Applied Psychology. All subjects gave written informed consent in accordance with the Declaration of Helsinki. The participants received a T-shirt after participating in the study. There were no defined exclusion criteria.

In total, 48 men (*M*_age_ = 22.52, *SD*_age_ = 4.55) participated in the described part of the study^1^. The lack of female participants is a consequence of the gender structure of the firefighting profession. Only a small number of women were enrolled in the aforementioned school and worked in the firefighting units. They were not drawn for the study described in the present paper.

The participants were randomly assigned to one of the conditions (23 in the experimental condition with virtual bystanders and 25 in the control condition without such bystanders). There was no age difference [*t*_(46)_ = 1.08, *p* = 0.284] between conditions. None of participants reported problems with perception of VR, and all of them had previously learned to control the simulator (they participated in two previous iterations of the study where the same simulator was used and they were also instructed about all possible actions and commands shortly before the experimental task).

### 2.2. Procedure

**Figure 1:**
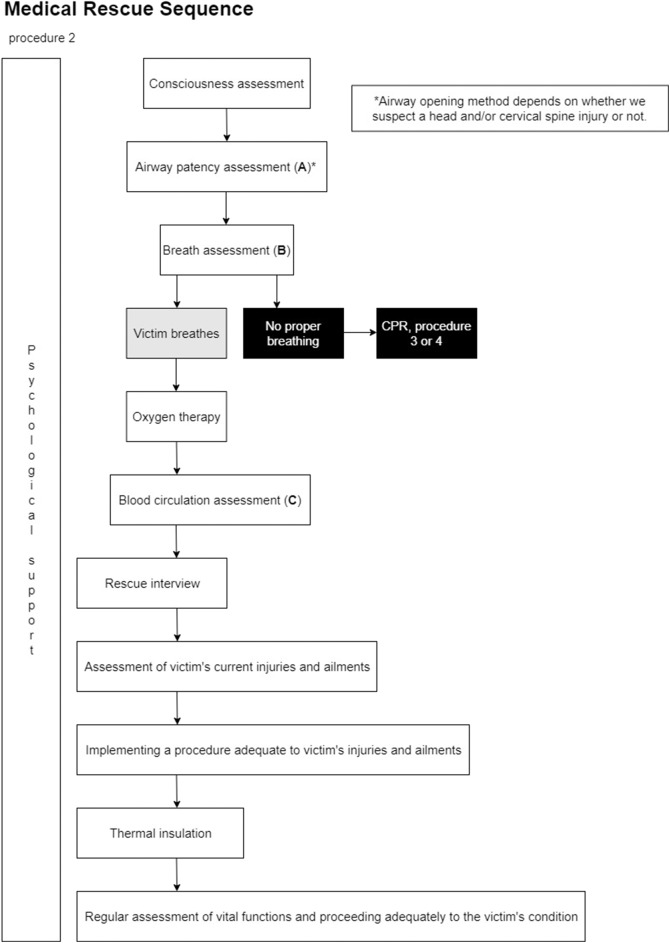
The medical rescue sequence used for the experimental task in the study.

Firstly, participants were briefly interviewed and equipped with apparatus for measuring physiological variables (ECG, ICG, EDA)^2^. Directly before the task started, the participants were informed that they would be asked to perform the Medical Rescue Sequence detailed in the National Firefighting Rescue System documentation^3^ For the full description of the procedure (see Figure 1). The task had a fixed 5 min duration. The experimenter received a confirmation of knowledge of the procedure from each participant.

**Figure 2:**
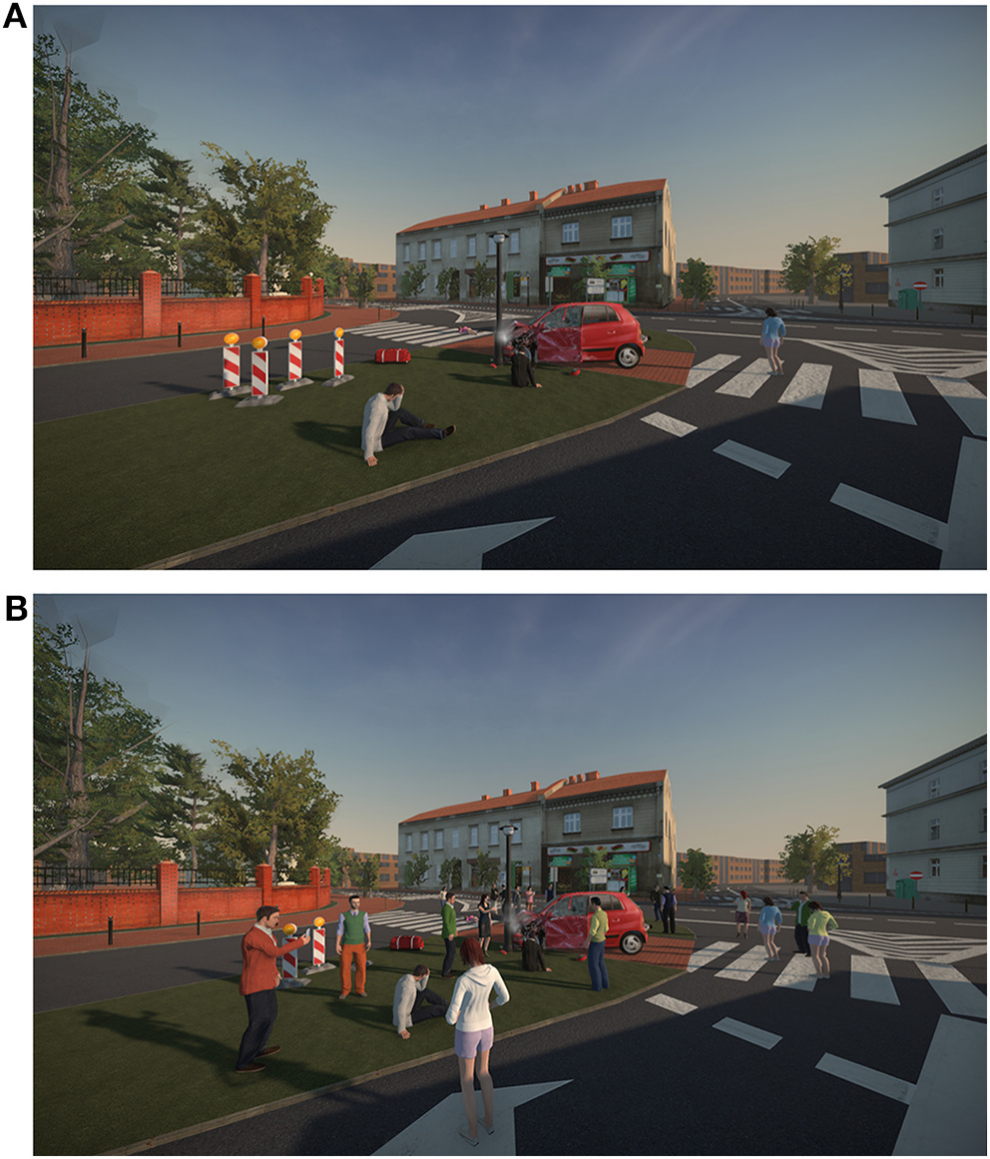
Screenshots showing the virtual environment used in the control **(A)** and experimental condition **(B)**. This point of view was available for the participants.

**Figure 3:**
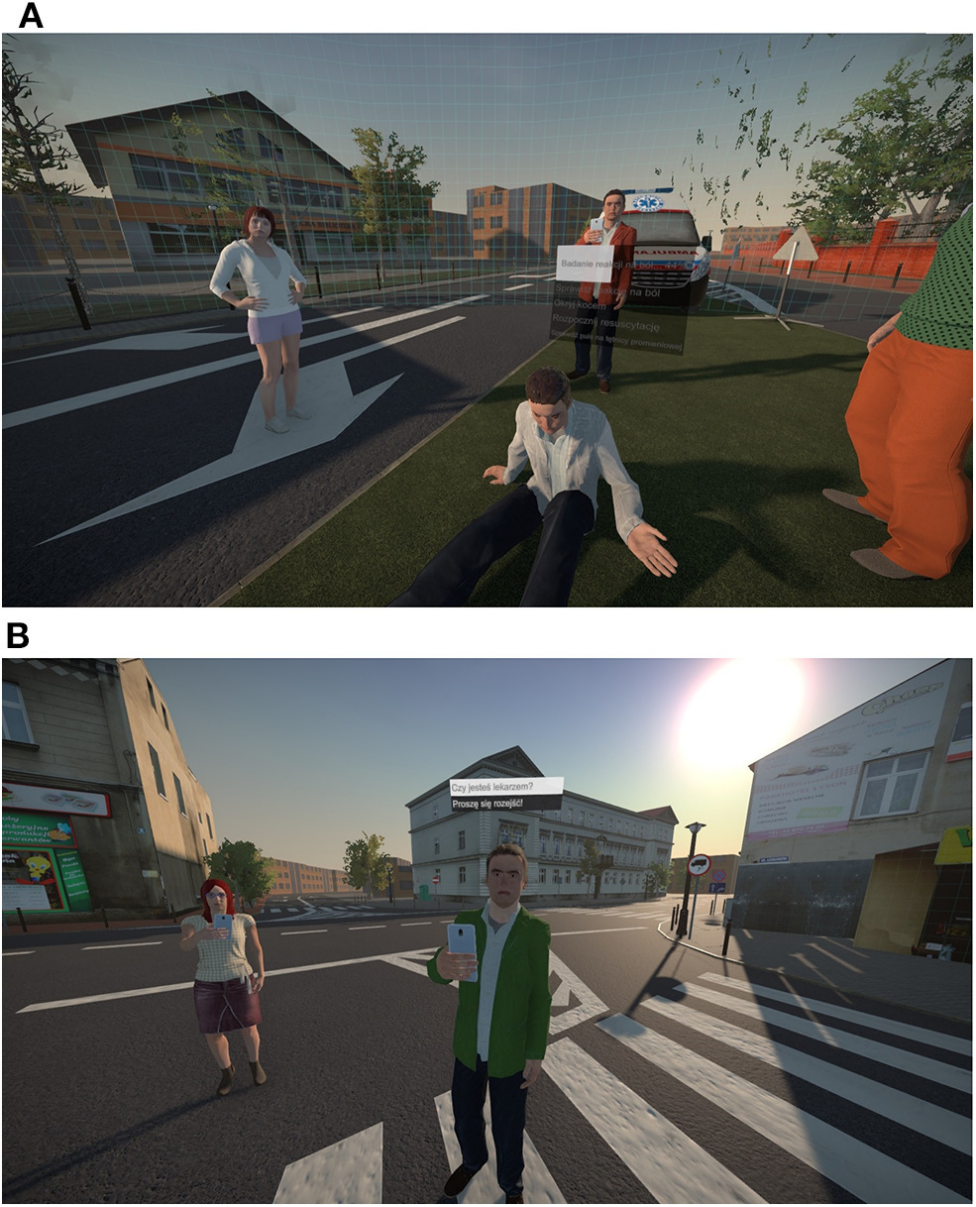
Screenshots showing the context menu in use: for a victim, during an action **(A)** and for a bystander, before choosing an action **(B)**.

Then, the VR simulation took place. Participants wore a HTC Vive head-mounted display (HMD) with hand-held controllers and headphones. The HMD was connected to a PC with a 3.40 GHz Intel Core i7 processor, 16 GB of RAM and a NVIDIA GeForce GTX 1080 graphics card. The simulation was developed with use of the Unity engine and depicted a collision between a car and a group of six pedestrians on an intersection in a small town (see Figure 2A). The possible interactions with victims were: conducting the SAMPLE interview^4^, checking several physical parameters (pulse, pain reaction, breathing, airways, and capillary recurrence), covering the person with a blanket, dressing the wounds, performing resuscitation. Moreover, passive oxygen therapy could have been performed with the equipment from the medical bag. All actions were controlled with text commands in a context menu. The menu consists of a list of possible actions, which could be accessed when pointing with a hand-held controller at a specific virtual agent and pushing one of the buttons. To choose between the available actions, the participant had to scroll on a trackpad of the controller. For an example of the menu interface for a victim (see Figure 3A). For photos of the experimental setup see the Supplementary Materials, and for a video with an example of actions conducted in the simulator used in the study see: https://www.youtube.com/watch?v=Bn_E4wX1RPE&feature=emb_logo.

The experimental condition contained an additional group of three bystanders located next to each victim (18 virtual agents in total, see Figure 2B). The bystanders were animated: they performed simple gestures at random moments, followed the participant with their eyes and some of them recorded the event with smartphones. They could be asked (through the list of actions in the menu) whether they were a doctor (and always responded “No”) and they could be told to move away (what they always did when asked to). For an example of the menu interface for a bystander (see Figure 3B).

For safety reasons, the experimenter was present in the room during the simulation, but she remained silent and could not be seen by participants. After the experimental task, the participants completed questionnaires administered using a PsychoPy script (Peirce, 2007, 2009).

### 2.3. Measures

The questionnaires used in this study were completed in polish language. The Polish versions were created on basis of the back translation procedure (Brislin, 1970). First of all, two independent German-speaking professional translators translated all of the items from German to Polish. In the next step, two different translators translated back into German. Then, we compared the original versions with those obtained during translation procedure. There were no major discrepancies between them.

#### 2.3.1. Manipulation Check

To assess whether participants noticed the bystanders, we asked them whether they perceived the following elements of the environment: a dog, a drone, policemen, a toy, and bystanders (critical question). Some of them were stimuli in other experimental conditions and some of them were masking items.

#### 2.3.2. Co-presence

The Polish version of The Co-Presence and Social Presence in Virtual Environments Scale (C-PS, Poeschl and Doering, 2015) was administered after the VR session to measure co-presence. It consists of four factors: Reaction to Virtual Agents (4 items), Perceived Virtual Agents' Reaction (4 items), Impression of Interaction Possibilities (4 items), (Co-)Presence of Other People (3 items). The items are rated on a 5-point Likert scale from −2 to 2. We evaluated internal consistency using the reliability analysis. The obtained Cronbach's coefficient was high (α = 0.89).

#### 2.3.3. Sense of Presence

The Polish version of 14-item iGroup Presence Questionnaire (IPQ, Schubert et al., 2001) was used to assess sense of presence. It contains 14 items on three subscales: (1) Spatial Presence (6 items), (2) Involvement (4 items), and (3) Realism (4 items). All of the items are rated on 7-point Likert scale from −3 to 3; overall score ranges from −42 to 42. We evaluated internal consistency using the reliability analysis. The obtained Cronbach's coefficient was satisfying (α = 0.77).

#### 2.3.4. Realism

The German VR Realism Scale in Polish version (VRRS, Poeschl and Doering, 2013) was used to assess perceived realism of simulation. In total it consists 14 items rated on 5-point Likert scale from −2 to 2; overall score ranges from −28 to 28. Thirteen items are divided on three subscales: Scene Realism (5 items), Audience Behavior (4 items), and Audience Appearance (4 items), remaining one item regards sound realism. We evaluated internal consistency using the reliability analysis. The obtained Cronbach's coefficient was very high (α = 0.92).

#### 2.3.5. Performance and Activity

To quantify performance, we developed a script automatically logging the correctness of actions taken during the exercise on a basis of National Firefighting Rescue System documentation. Since the rescue procedure we used is defined in the form of an algorithm, we could precisely determine the correctness of the participants' actions. In order for each individual action to be considered “correct,” it had to be taken exactly when the rescue procedure foresees it. Otherwise (e.g., performing an unforeseen action or confusing the order), the single action was considered an “error.” We counted all actions taken in the wrong order (“errors”). No feedback on the performance was given during the session. An erroneous action could not be corrected, but further actions were calculated according to the rule presented above—it was feasible to avoid further mistakes simply by performing subsequent actions in the correct order resulting from previous decisions.

Moreover, it was possible to count the total sum of taken actions, regardless of their correctness. Therefore, such index was calculated in order to test the possible impact of audience presence on activity.

#### 2.3.6. Other Measures

Participants completed the Polish versions of several other questionnaires at the end of the study: Self-Assessment Manikin (SAM, Bradley and Lang, 1994), The Scale of Emotions (Wojciszke and Baryła, 2005), The Stress Appraisal Questionnaire (SAQ, Włodarczyk and Wrześniewski, 2010), Simulator Sickness Questionnaire (SSQ, Kennedy et al., 1993), The Scale of Aesthetics (Chevalier et al., 2014), NASA Task Load Index (NASA-TLX, Hart and Staveland, 1988; Zieliński and Biernacki, 2010). These tools are not of interest to the hypotheses formulated in the present paper, therefore the analyses concerning the aforementioned variables will not be reported herein.

### 2.4. Data Analysis

#### 2.4.1. Null Hypothesis Significance Testing and Equivalence Testing

For a proper interpretation of the results it was decided to firstly exclude that the variability in data stems from sources other than the social facilitation effect. Performance could vary between groups not only because of the social facilitation effect, but also because of differences in terms of the mere number of actions conducted by the participants. Therefore, it was checked whether the number of actions in the groups is statistically equivalent. In such cases equivalence testing (two one-sided *t*-tests—TOST) is used (Limentani et al., 2005; Lakens et al., 2018, 2020). For other hypotheses, null hypothesis significance testing (NHST) was used.

#### 2.4.2. Moderation Analysis

**Figure 4:**
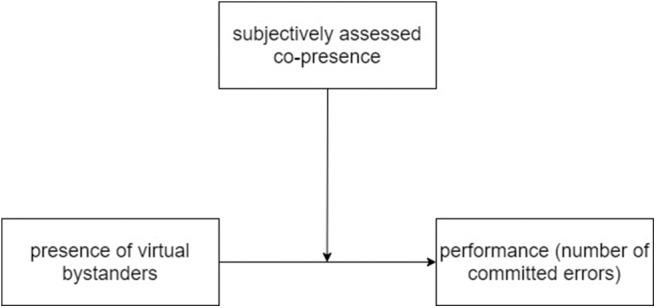
Graphical representation of the moderation analysis (Hayes, 2013). Other tested moderators (sense of presence and perceived realism) could be put in place of co-presence in this visualization.

For the verification of research hypotheses, moderation analysis was chosen to be used. Such analysis tests the influence of a third variable (moderator) on the relationship between independent and dependent variables. Moderation analysis is used to answer the question which conditions have to be met for an effect to occur (see Figure 4 for an example of conceptualization of moderation). It is calculated based on a regression model. In the case of the present analysis, the model is as follows:

(1) $$\begin{array}{r} performance=b_{0}+b_{1condition}+b_{2moderator}+b_{3\left(condition*moderator\right)} \end{array}$$

Moderator can enhance, reduce or change the influence of predictor on the outcome variable. In moderation analysis with a single moderator (as in the case of the reported study), three main effects are calculated: the separate influences of the predictor and moderator and the interaction of these variables (Fairchild and MacKinnon, 2009).

If the interaction effect is significant, simple main effects are calculated. Such effects indicate on which level of the moderator the influence exists. In the PROCESS macro (Hayes, 2013), subsamples for simple main effects can be chosen with thresholds of +/− 1 SD or 16th, 50th, 84th percentiles on the moderator. The latter technique was used to differentiate between high and low levels of considered moderators in the reported analyses. Additionally, the Johnson-Neyman technique can be used in order to determine the region of significance. This technique is also useful for preparing data for visualization (Johnson and Fay, 1950; D'Alonzo, 2004). Both these techniques will be used in the present paper.

Standardized effect sizes for the moderation analysis were calculated according to Bodner's (2017) guidelines. Thanks to such approach, effect sizes in separate analyses can be compared in terms of strength. Standardized effect sizes higher than 0.4 and lower than 1.0 are considered *small*, higher than 1.0 and lower than 1.6 are *medium* and higher than 1.6—*large*.

## 3. Results

Data were analyzed with Imago Pro 5.0, the PROCESS (Hayes, 2013) macro and the R environment—the TOSTER package (Lakens, 2017). Data from four participants were excluded due to technical problems with performance logging. Finally, data from 44 participants (22 in each group) were analyzed. For the analyses, the control group was dummy coded as 1 and the experimental group was dummy coded as 2.

### 3.1. Manipulation Check

The manipulation was successful, only 5 out of 22 participants in the experimental condition reported that they did not notice the bystanders, and 5 out of 22 participants in the control condition reported they noticed bystanders when they could in fact not see them.

### 3.2. Audience Impact on Activity

To check the possibility that audience presence affected participants' activity (the number of actions), we compared both conditions in such terms. We found that the difference in number of actions in both conditions was not statistically significant and slightly above the “medium” threshold in terms of effect size. (*M*_control_ = 25.32, *SD*_control_ = 8.29, *M*_audience_ = 21.27, *SD*_audience_ = 6.73, *t*(42) = 1.78, *p* = 0.08, *d* = 0.53).

Because the *t*-test yielded insignificant results, we applied the TOST procedure (Limentani et al., 2005; Lakens et al., 2018, 2020). We determined the smallest effect size of interest (SESOI) on the basis of results obtained in the previous iteration in the longitudinal study. Using the study's alpha level and sample size, we calculated the critical effect size (Cohen's *d*, Cohen, 1992). The equivalence test was non-significant, *t*_(42)_ = 0.320, *p* = 0.625, given equivalence bounds of −0.44 and 0.44 and an alpha of 0.05. Based on both the equivalence test and the null-hypothesis test, we may conclude that the observed effect is statistically not different from zero and statistically not equivalent to zero.

### 3.3. Audience Impact on Performance

In order to test the main effect of audience presence on performance, we separately compared both conditions in terms of errors made. We found main effect of bystanders presence on performance to be statistically not significant and small in terms of effect size (*M*_control_ = 9.73, *SD*_control_ = 3.71, *M*_audience_ = 8.27, *SD*_audience_ = 3.27, *t*_(42)_ = 1.38, *p* = 0.175, *d* = 0.42). Thus, we did not observe the main effect of social facilitation, it is possible the effect was to small to met conventional criterion of alpha 0.05 with the sample size we used.

### 3.4. Interaction of Perceived VR Characteristics and the Presence of Agents in the Performance

We conducted moderation analysis according to the steps described in *Data Analysis* section. None of the considered moderators affected the performance on its own (see Table 1 for main effects of hypothesized moderators on performance). Also, *t*-test was conducted to evaluate the influence of audience presence on the considered moderators—co-presence, sense of presence and realism. The analysis did not reveal the significant effects (see Table 2).

**Table 1:** Simple linear regression results for considered moderators of performance (social facilitation).

**Predictor**	**β**	***p***	**95 % CI**^*******a*******^		***R^**2**^***
**Co-Presence**	−0.006	0.968	−1.76	1.69	<0.01^*^
C-PS: Reaction to virtual agents	−0.012	0.939	−1.17	1.08	<.01^*^
C-PS: Perceived virtual agents' reaction	0.197	0.200	−0.49	2.26	0.04
C-PS: Impression of interaction possibilities	−0.041	0.791	−1.48	1.13	<0.01^*^
C-PS: co-presence of other people	−0.221	0.148	−2.49	0.39	0.05
**Sense of presence**	−0.065	0.674	−0.50	0.33	<0.01^*^
IPQ: Spatial presence	−0.068	0.662	−1.31	0.84	<0.01^*^
IPQ: Involvement	0.084	0.589	−1.22	0.70	<0.01^*^
IPQ: Realism	−0.007	0.964	−0.95	0.91	<0.01^*^
**Realism**	−0.041	0.791	−1.75	1.34	<0.01^*^
VRRS: Scene realism	0.068	0.663	−1.19	1.85	<0.01^*^
VRRS: Audience behavior	−0.059	0.704	−1.51	1.03	<0.01^*^
VRRS: Audience appearance	−0.118	0.446	−1.66	0.74	0.01
VRRS: Sound realism	−0.022	0.889	−1.29	1.12	<0.01^*^

* *R^2^ < 0.01—predictor explains <1% of variances*.

a *95% Confidence interval*.

**Table 2:** *T*-test results comparing the experimental and control group on considered moderators.

**Predictor**	**Experimental**		**Control**		***t*****-test**			
	***M***	***SD***	***M***	***SD***	***t***	***df***	***p***	***d***
Co-presence	−0.52	0.55	−0.29	0.71	1.22	42	0.230	0.37
Sense of presence	−0.28	0.90	−0.18	0.88	0.38	42	0.702	0.12
Realism	0.14	0.68	0.08	0.75	0.29	42	0.777	−0.09

#### 3.4.1. Co-presence as a Moderator of Audience Presence and Performance Relationship

**Figure 5:**
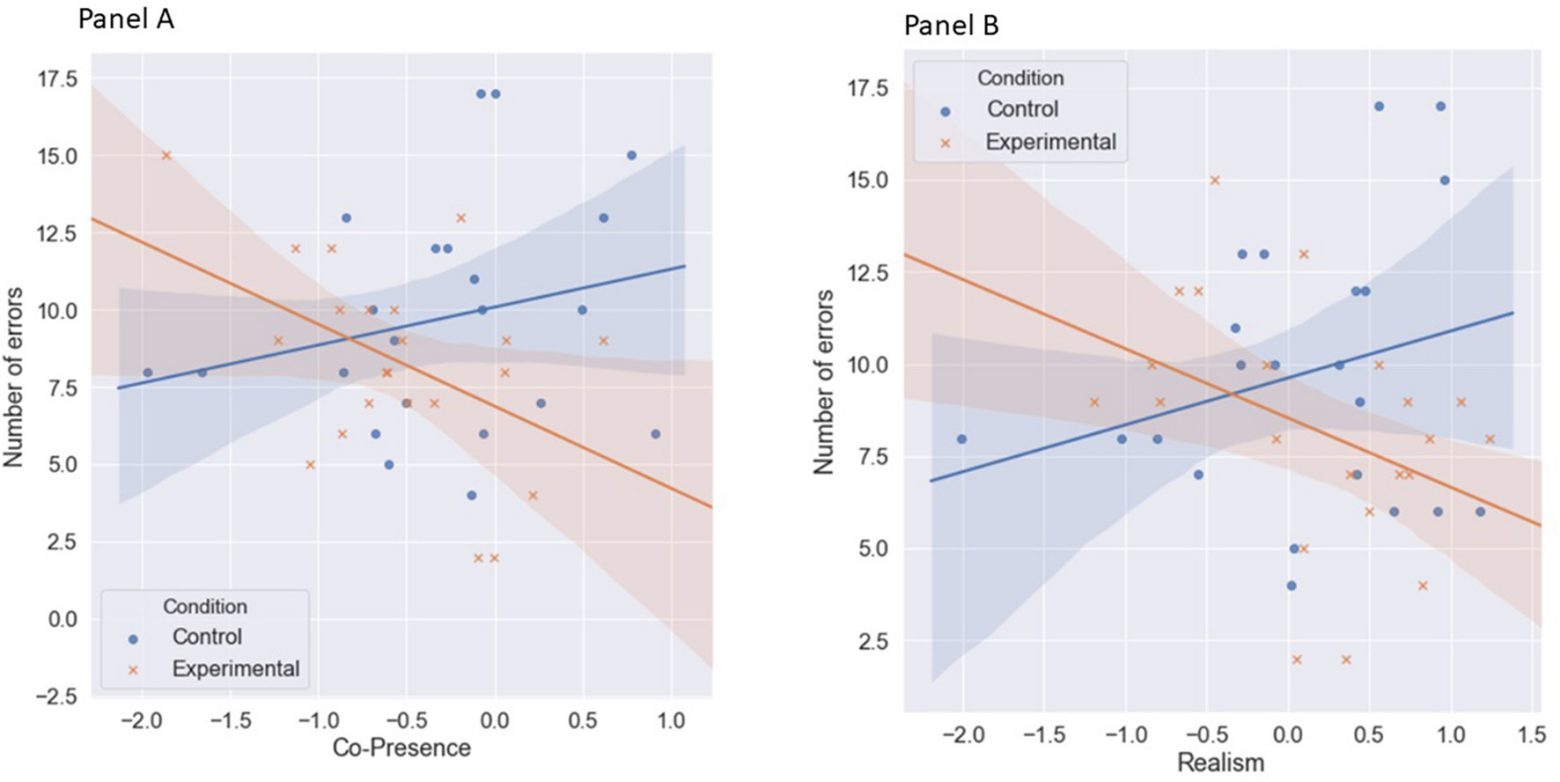
Graphical representation of the interaction—the number of errors committed in VR simulation depending on the interaction of the presence of audience and the subjectively assessed VR characteristics: co-presence **(A)** and sense of realism **(B)**.

According to our first hypothesis, we analyzed the interactional influence between audience presence and subjectively assessed co-presence on performance. We found a statistically significant interaction (β = −2.48, *b* = −3.88, *SE* = 1.68 *p* = 0.026, *r*^*2*^_increase_ = 0.11). Calculated simple main effects indicated that only in the case of high assessment of co-presence did the audience cause social facilitation and the effect was of moderate strength (β = −4.08, *SE* = 1.52, *p* = 0.010, *95% CI:* −7.15, −1.02, δ = −1.22). For co-presence assessed as low, the relationship was insignificant and the effect size was very small (β = 0.42, *b* = 0.42, *SE* = 1.33, *p* = 0.75, *95% CI:* −2.26, 3.10, δ = 0.13). This dependency is shown in Figure 5A. Additionally, we used the Johnson-Neyman technique to determine the specific values of co-presence at which the moderation occurred. We found that relatively high (higher than −0.269) co-presence levels resulted in social facilitation in the presence of bystanders. Above that threshold scored 19 participants (11 in control group and 8 in experimental group).

Moreover, we decided to check whether specific subscales are moderators of described dependency. We found two subscales of Co-Presence (Perceived Virtual Agents' Reaction and Impression of Interaction Possibilities) to be statistically significant moderators (see Table 3 for all interaction results). Accordingly to Hypothesis 1, we found an interactional effect of audience presence and subjectively assessed co-presence on performance (social facilitation).

**Table 3:** Interaction between subjectively assessed VR characteristics and the presence of agents on performance.

**Predictor**	**β**	***b***	**SE**	***t***	***p***	**95%CI**	
**Co-Presence x Condition**	−2.48	−3.88	1.68	−2.31	0.026^*^	−7.28	−0.49
Reaction to virtual agents × Condition	−1.02	−1.04	1.17	−0.89	0.378	−3.41	1.33
Perceived virtual agents' reaction × Condition	−2.24	−2.85	1.39	−2.05	0.047^*^	−5.66	−0.04
Impression of interaction possibilities × Condition	−2.23	−2.66	1.31	−2.02	0.049^*^	−5.32	−0.001
(Co-)Presence of other people × Condition	−1.80	−2.42	1.49	−1.62	0.113	−5.44	0.60
**Sense of presence** **×** **Condition**	−0.64	−0.24	0.41	−0.59	.556	−1.07	0.59
Spatial presence × Condition	0.14	0.12	0.97	0.13	0.900	−1.84	2.09
Involvement × Condition	−0.58	−0.57	1.09	−0.53	0.601	−2.77	1.62
Realism × Condition	−1.28	−1.08	0.95	−1.15	.259	−3.00	0.83
**Realism** **×** **Condition**	−2.24	−3.15	1.46	−2.15	0.037^*^	−6.11	−0.19
Scene realism × Condition	−1.36	−1.88	1.55	−1.22	0.230	−5.01	1.24
Audience appearance × Condition	−2.17	−2.38	1.15	−2.07	0.044^*^	−4.71	−0.06
Audience behavior × Condition	−2.23	−2.57	1.99	−2.14	0.038^*^	−4.99	−0.15
Sound realism × Condition	−1.48	−1.62	1.22	−1.33	0.189	−4.08	0.84

* *p < 0.05*.

#### 3.4.2. Sense of Presence as a Moderator of Audience Presence and Performance Relationship

According to our second hypothesis, we analyzed the interactional influence between audience existence in the scene and subjectively assessed sense of presence on performance. The analysis ruled out the role of this variable as a moderator, due to the statistical insignificance of the results (β = −0.64, *b* = −0.24, *SE* = 0.41 *p* = 0.556, *r*^*2*^_increase_ = 0.01).

#### 3.4.3. Sense of Realism as a Moderator of Audience Presence and Performance Relationship

According to our third hypothesis, we analyzed the interactional influence between audience existence in the scene and subjectively assessed sense of presence on performance. We found a statistically significant interaction (β = −2.24, *b* = −3.15, *SE* = 1.46 *p* = 0.037, *r*^*2*^_increase_ = 0.10). Calculated simple main effects indicated that high level of realism resulted in statistically significant, positive relationship between virtual agents' presence and subjects' performance—social facilitation, with a moderately strong effect size (β = −3.79, *SE* = 1.49, *p* = 0.015, *95% CI:* −6.80, −0.77, δ = −1.12). For low perceived realism, the relationship was insignificant and the effect size was very small (β = 0.90, *SE* = 1.49, *p* = 0.55, *95% CI:* −2.12, 3.92, δ = 0.27). This dependency is shown in Figure 5B. Then, we used the Johnson-Neyman technique in order to determine the specific values of realism at which the moderation occurred. We found that relatively high and positive (higher than 0.347) realism levels resulted in social facilitation in the presence of virtual bystanders. Above that threshold scored 19 participants (10 in control group and 9 in experimental group).

Moreover, we decided to check whether specific subscales are moderators of the described dependency. We found two subscales: Audience Appearance and Audience Behavior to be statistically significant moderators (see Table 3 for interaction analysis results).

## 4. Discussion

The current results provide support for the moderating role of co-presence (Hypothesis 1) and realism (Hypothesis 3) on social facilitation in VR. On the other hand, sense of presence did not play the same role (Hypothesis 2). In order to successfully evoke the social facilitation effect in the presence of computer-generated bystanders, a certain level of (subjective) co-presence in the VE must be achieved. Realism plays an analogous role. In this study, mere presence of virtual agents on the simulated accident site was enough to improve the trainees' performance (reduce the number of erroneous actions), but only for those who evaluated co-presence and realism as relatively high.

We found no main effect of bystanders' presence on performance, which is in line with some previous studies (Hayes et al., 2010; Baldwin et al., 2015; Pan and Hamilton, 2015). Although the main effect of social facilitation seemed to be statistically insignificant, the effect sizes are of medium strength. Perhaps, a non-moderated social facilitation occurs in VR but it is more difficult to detect than in the real world. In this regard, our results may shed new light on the inconclusiveness of previous studies in which social facilitation did not always occur —it is possible that social realism (co-presence and realism) was not taken into account there. We can only speculate that, in some cases, social facilitation might have taken place, but only among participants who experienced high social realism in the VE. Not only the results for high levels of moderators were statistically significant, but the observed effects were of medium strength (−1.22 for co-presence and −1.12 for sense of realism). At the same time, the effects for low levels of moderators were insignificant and very weak (0.13 for co-presence and 0.27 for sense of realism). This can be interpreted as consistent with the general theory regarding social facilitation, which assumes that mere presence of real actors is enough for evoking the effect. Since the vast majority of previous experiments was conducted in the presence of real observers, it can be assumed that they were perceived as real and interactive. Moreover, some theorists emphasized the importance of establishing a basic psychological relationship between the actor and the observer, claiming that mere physical presence is insufficient also in the real world (Cottrell et al., 1968; Cottrell, 1972). Moreover, no physical presence (even symbolic) is needed to produce the effect: for example, social facilitation takes place in online auctions and the level of symbolic presence plays a role in the absence of a physical presence in this case (Rafaeli and Noy, 2002).

The above considerations are consistent with the results we obtained in exploratory analyses described earlier. Since we inspected the role of individual subscales, we were able to identify those which played the crucial role in the interactions we predicted —note the Realism scale we used includes four aspects of realism—scene, sound, audience appearance and audience behavior. As it turned out, only the last two of them, exactly those related directly to social context, played a role in the moderation we found. Findings regarding Co-Presence are also consistent. In this case two subscales directly related to social interactions played a similar role. The whole picture seems to be complemented by the fact that the third scale (Presence, regarding the subjective sense of presence), which did not turn out to moderate social facilitation, does not include any subscale related to social interactions. Keeping in mind the exploratory nature of these results, further detailed research is needed, but these results clearly suggest the crucial role of social realism in evoking phenomena based in social interactions in VR.

Our results should be also analyzed from the perspective of the new terminology of phenomena regarding illusion of VR realism proposed by Slater (2009). The idea of two orthogonal components of realistic response to VR seems to fit well with our results. Slater proposed the term “place illusion” (PI) as the name of qualia of “being there” which, accordingly to Slater's conclusions, is rather of perceptional than cognitive nature, it is often called “Presence.” The second dimension called “plausibility illusion” (Psi) refers to the illusion that events being depicted are actually occurring. It is more difficult to achieve and more susceptible to being broken. Both components are needed to evoke reactions similar to expected in reality called “response-as-if-real” (RAIR). In the case of the present study social facilitation was the RAIR. From this perspective, one could notice that variables we tested as moderators may be assigned to PI or Psi. Presence undoubtedly belongs to PI. Also two aspects of Realism (scene and sound) are rather of perceptual nature while audience behavior should rather be seen as belonging to the Psi domain. Only audience appearance may seem to be difficult to classify, but looking carefully at items (e.g., “Virtual humans in their entirety seemed to be authentic for this occasion.”), one can see that they place a lot of emphasis not so much on the appearance itself as on the adequacy of the appearance to the situation, which may suggest assigning this subscale to Psi also. Attempt to classify Co-presence subscales could also be made—inspection of items may suggest that three of four would rather belong to Psi domain (Perceived Virtual Agents' Reaction, Impression of Interaction Possibilities, and Reaction to Virtual Agents) and the last one would be difficult to classify (Co-Presence of Other People). Remembering that the above reasoning was made post-factum, it is easy to see that the role of moderators of the relationship between the actual presence of observers on the stage and performance was played only by variables assigned to Psi. In other words RAIR depended on the sufficiently high level of Psi-related variables. In this light our findings may suggest that Psi may be particularly important in evoking social reactions involving high-order cognitive processes.

Our results may contribute to the theoretical dispute about the mechanism of the social facilitation effect. According to one of the most popular explanations, attentional conflict caused by the physical presence of other people is the basis of the social facilitation phenomenon (Baron, 1986). If this is true in the case of VR, one could expect social facilitation to occur in VR training simply due to the existence of (social) distractors. Thus, the effect should occur regardless of the level of the subjective social realism, although it was not observed in our study. On the contrary, we found this effect to be dependent on the perceived level of co-presence and realism of the virtual agents. Therefore, it seems that Baron's explanation does not entirely match our results, according to which the distractors do not only need to *exist*, but they have to be perceived as *realistic* and *(co-)present* as well for the social facilitation effect to occur.

All of the aforementioned observations lead to a conclusion that it is useful to implement social stimuli in VR training simulators, especially in the case when real people are present during actual implementation of the task being trained. It would be beneficial to implement virtual agents in the simulation, as it was confirmed that such agents can, to some extent, evoke effects similar to those observed in the real world. Bystanders are very often present at accident sites. They influence the rescuers' emotions and may sometimes actively hinder the operation (as reported by the firefighters themselves; Strojny et al., 2018), therefore they should also be included in training procedures somehow. Moreover, while designing training simulators, attention should be paid to increasing the experience of social realism, e.g., through implementing realistic animations or a possibility to interact with the virtual agents. Moreover, social realism could be further increased by creating more diverse groups of bystanders. Such variations should also be tested in further studies.

There are several limitations to our study. We tested only the positive side of the effect (social facilitation in contrast to social inhibition). Since we conducted our study on specific participants, we could not find the inhibition effect during a task which was easy for them. However, using an existing procedure instead of an abstract task may increase the ecological validity of the study. Subsequent research should address this issue either by recruiting participants from a general population (not familiar with rescue procedures) or by manipulating the task in order to transform a well-practiced procedure into a counter-intuitive one. In both cases we would expect the social inhibition effect. Moreover, the characteristics of the population from which the participants were recruited led to the lack of representation of women in the study, which also may be viewed as a limitation of the study. Therefore, further studies with a more diverse group of participants should be conducted in order to improve generalizability of the results.

The hypotheses we formulated regarded all of three potential moderators separately. However, it is plausible that the moderators are interrelated—we did not take it into consideration during experiment preparation. A model that includes three moderators at the same time would require a much larger sample to draw conclusions from it. We see our results as a first step in analysing the phenomenon of social facilitation in VR in context of subjective perception of the virtual environment (i.e., co-presence, sense of presence, or realism). Testing whether these variables are related to each other should be considered in further studies on this issue.

Besides, we operationalized VR characteristics as self-reported. Further studies should use experimental manipulation of these variables. Moreover, we used a simple method of assessing the participants' performance —namely, the number of committed errors. In further studies it could be useful to develop a more sophisticated performance measure (e.g., not only the correctness but also the speed). Moreover, we used a well-documented, but still highly specific activity. In future studies more general tasks should be used to strengthen the external validity of the results.

Lastly, it could be viewed as a big limitation of the study that virtual agents—the victims—were present on the scene in both conditions. The understanding of social facilitation effect as the influence of *the mere presence* of other people, as it was firstly defined by Zajonc (1965) and showed in some studies (e.g., Markus, 1978; Platania and Moran, 2001) may lead to expectation of social facilitation occurrence in both conditions. In this case, due to design of the scene in our study we cannot draw conclusions about social facilitation. *Some* virtual people were *merely present* on the scene regardless of the condition. Adding the virtual bystanders would be viewed in this case as a change in *quantity*, not *quality* of the stimuli. However, mere presence might not be enough for evoking the effect, as it was proposed by Cottrell et al. (1968). In his study, mere presence of other people (not interested in the person performing the experimental task, not looking at them etc.) did not evoke the effect, while presence of *audience* (people observing the participant and overly interested in them). Therefore, it may not be the fact that social facilitation is evoked by other people being there, but by the roles they have, the affective states they evoke in participants (i.e., anticipation of evaluation by the audience). This interpretation matches our results—the virtual bystanders were in fact similar to Cottrell et al's (1968) audience—their purpose was to observe the participant and even to “record” their actions with smartphones. Therefore, we decided to go with Cottrell's argumentation. However, more studies on this issue should be conducted, with focus on distinguishing between effects of mere presence and (evaluative) audience presence.

In sum, co-presence and realism seem to play an important moderating role in the relationship between the presence of computer-generated agents and social facilitation in VR. This finding is crucial considering the increasing use of similar tools to teach complex skills in a social context.

## Data Availability Statement

The datasets generated for this study are available on request to the corresponding author.

## Ethics Statement

The studies involving human participants were reviewed and approved by Ethical Committee at Jagiellonian University Institute of Applied Psychology. The patients/participants provided their written informed consent to participate in this study.

## Author Contributions

PS created the main conception and study design, analyzed majority of the data, interpreted the results, drafted and prepared the final version of the paper. NL and ND-M performed the experiment, gathered, and partially analyzed data. AS provided substantial contribution to the conception of the experiment and took part in data interpretation. ND-M wrote a minor part of the paper. NL visualized data. All authors contributed to manuscript revisions, read and approved the submitted version.

## Conflict of Interest

The authors were employed by company Nano Games sp. z o.o.

## Footnotes

^1^The current study is a part of longitudinal study with three experimental and one control group. For the purpose of this report, we present data from one of the experimental conditions and the control condition from the third iteration of the study. The other conditions were unrelated to social facilitation.

^2^Data gathered with this equipment is irrelevant to social facilitation and is discussed in another paper.

^3^Available in Polish at https://www.straz.gov.pl/download/1854.

^4^SAMPLE is an acronym for six basic questions in a medical assessment: symptoms, allergies, medications, past medical history, last oral intake, and events leading up to present injury.
