# Supplementary material for: Moderators of Social Facilitation Effect in Virtual Reality: Co-presence and Realism of Virtual Agents
Source: Front Psychol. 2020 Jun 15;11:1252. doi: 10.3389/fpsyg.2020.01252 (PMC7307460; doi:10.3389/fpsyg.2020.01252)

Supplementary Materials for the article „Moderators of Social Facilitation Effect in Virtual Reality: Co-presence and Realism of Virtual Agents”

The pictures presented below show the participants of the study during the experimental task.

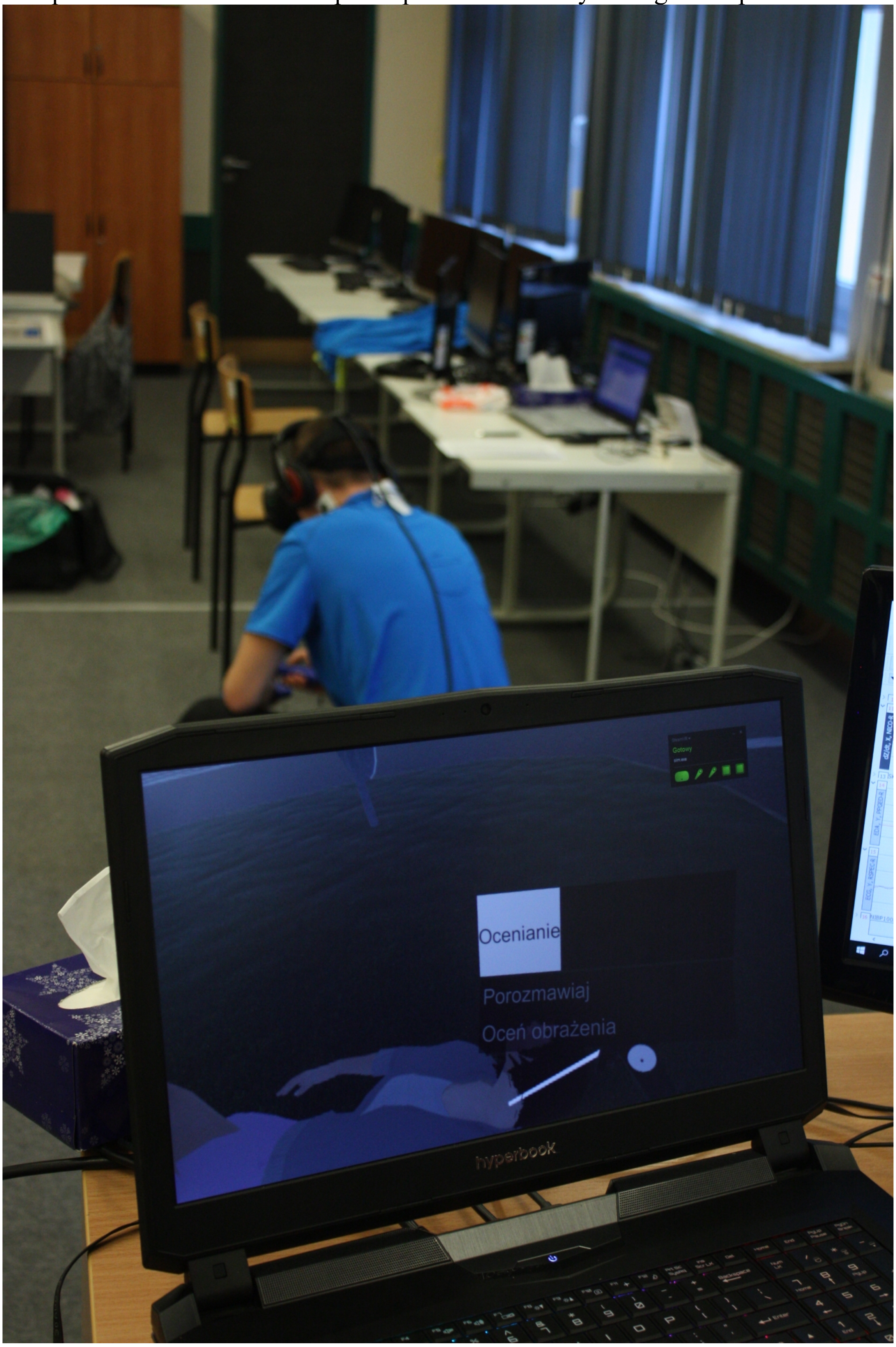

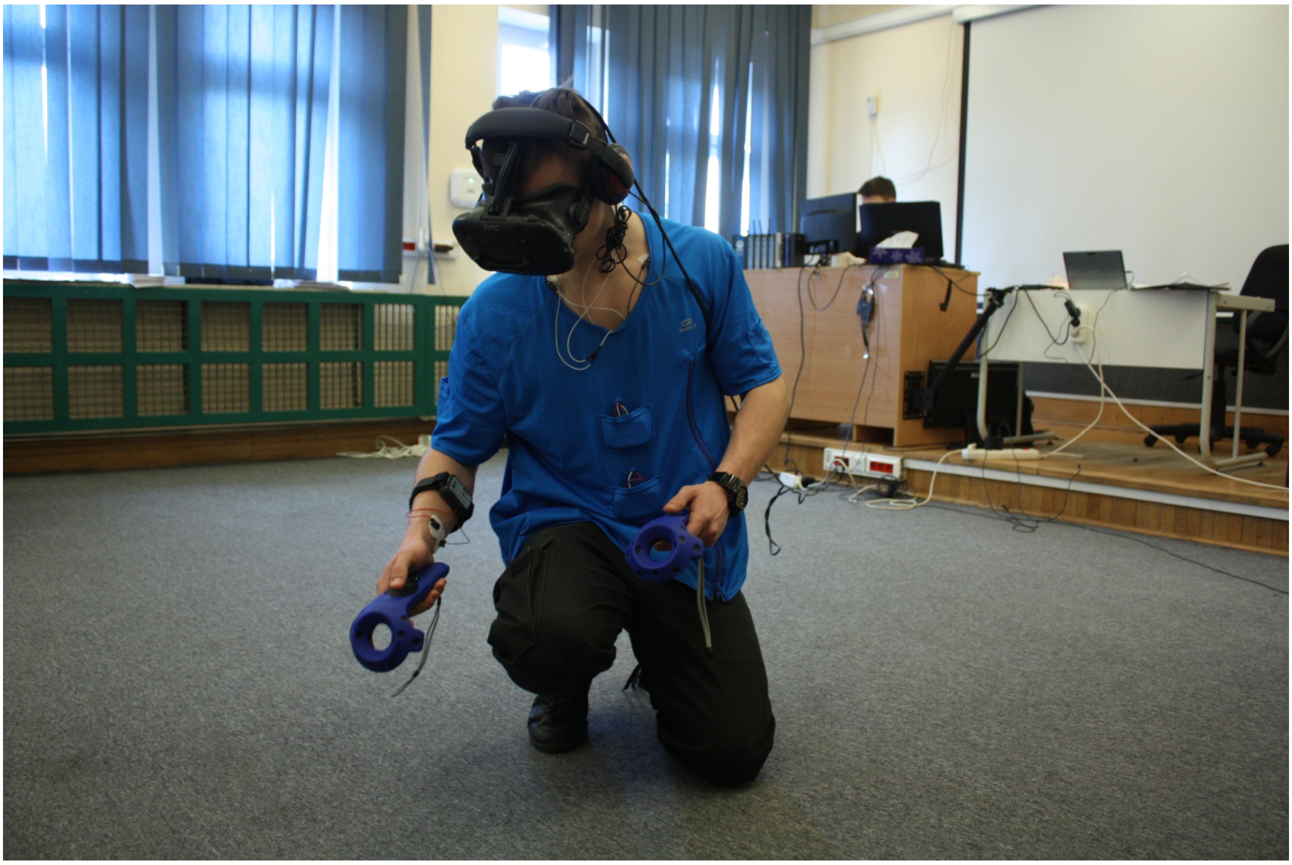

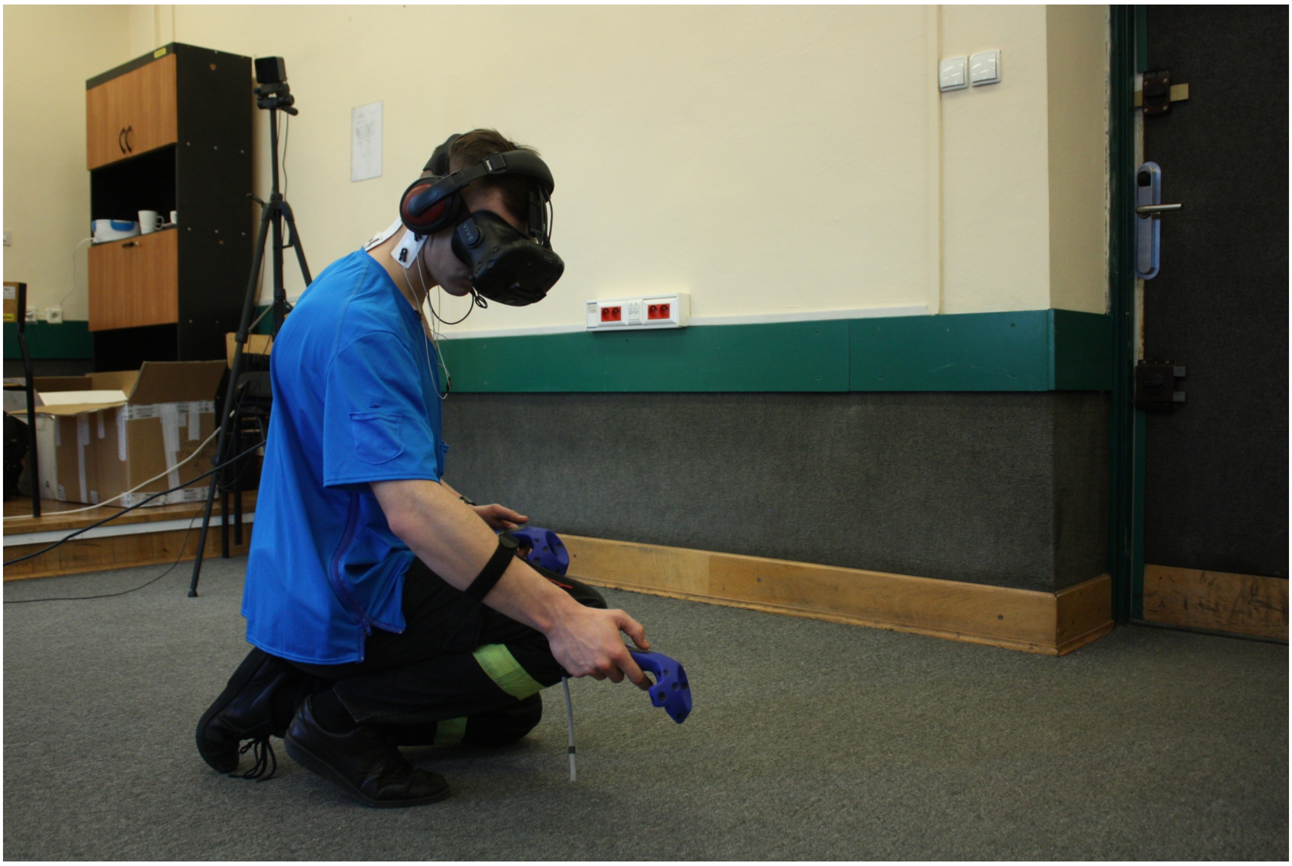

Supplement: Supplementary file 1 [file Image_1.PDF]
